# Leaf Anatomy, Morphology and Photosynthesis of Three Tundra Shrubs after 7-Year Experimental Warming on Changbai Mountain

**DOI:** 10.3390/plants8080271

**Published:** 2019-08-07

**Authors:** Yumei Zhou, Jifeng Deng, Zhijuan Tai, Lifen Jiang, Jianqiu Han, Gelei Meng, Mai-He Li

**Affiliations:** 1 Ecological Technique and Engineering School, Shanghai Institute of Technology, Shanghai 201418, China; 2 Department of Tourism Economy, Changbai Mountain Academy of Sciences, Baihe 133633, China; 3 Center for Ecosystem Science and Society, Northern Arizona University, Flagstaff, AZ 86011, USA; 4 Swiss Federal Research Institute WSL, Zuercherstrasse 111, 8903 Birmensdorf, Switzerland; 5 School of Geographical Sciences, Northeast Normal University, Changchun 130024, China

**Keywords:** anatomical traits, gas exchange, leaf size, open-top chamber, warming

## Abstract

Tundra is one of the most sensitive biomes to climate warming. Understanding plant eco-physiological responses to warming is critical because these traits can give feedback on the effects of climate-warming on tundra ecosystem. We used open-top chambers following the criteria of the International Tundra Experiment to passively warm air and soil temperatures year round in alpine tundra. Leaf size, photosynthesis and anatomy of three dominant species were investigated during the growing seasons after 7 years of continuous warming. Warming increased the maximal light-saturated photosynthetic rate (*P*_max_) by 43.6% for *Dryas. octopetala* var. *asiatica* and by 26.7% for *Rhododendron confertissimum* across the whole growing season, while warming did not significantly affect the *P*_max_ of *V. uliginosum*. The leaf size of *Dr. octopetala* var. *asiatica* and *Rh. confertissimum* was increased by warming. No marked effects of warming on anatomical traits of *Dr. octopetala* var. *asiatica* were observed. Warming decreased the leaf thickness of *Rh. confertissimum* and *Vaccinium uliginosum*. This study highlights the species-specific responses to climate warming. Our results imply that *Dr. octopetala* var. *asiatica* could be more dominant because it, mainly in terms of leaf photosynthetic capacity and size, seems to have advantages over the other two species in a warming world.

## 1. Introduction

Global climate change causes non-uniform warming, with faster and stronger warming in high latitudes and altitudes where tundra exists [1]. Tundra develops at low temperatures, and air warming will affect many other environmental factors such as moisture and nutrient availability, and thus directly or/and indirectly influences eco-physiological functions of plants [2]. Even a relatively small increase in temperature has caused strong responses of plants in the tundra ecosystem [3,4]. It has been expected that present and future warming could lead to pronounced effects on tundra plants. 

Many studies have reported phenology [5], shrub expansion [6], growth [7,8], gas exchange [9], morphological changes [10], and anatomical changes [11] in tundra plants in response to warming. Among these changes, the photosynthetic adjustment was found to be relatively rapid and sensitive to warming, while alterations in leaf morphology and anatomy showed adaptive responses to long-term warming.

Low temperature is one of the most important limiting factors determining the growth of alpine and tundra vegetation. Generally, photosynthesis increases with increasing leaf temperature until temperature reaches an optimum [12]. Therefore, the positive effect of warming on photosynthesis is expected for vegetation growing in the tundra. It has been widely reported that warming has increased photosynthetic carbon uptake for arctic tundra vegetation [13,14,15,16]. However, the magnitude and direction of photosynthetic responses of tundra plants to warming differ greatly with species. In-situ warming (+1.4 °C, 3 years) in the Antarctic has promoted photosynthetic assimilation in *Colobanthus quitensis* but not in *Deschampsia antarctica* [17]. The photosynthetic rate of *Picea glauca* was 41% greater in OTCs (+1.8 °C warming, three growing seasons) compared to that in the controls [18]. Knowledge of species-specific responses to warming can help to better understand and predict future changes in cover, abundance, distribution and adaptation of tundra plants in a changing world. 

Warming has been shown to induce morphological and anatomical changes in plants grown in a variety of environments including tundra. Long-term warming (+1~2 °C, 16 years) has increased leaf size and plant height but decreased specific leaf area in *Cassiope tetragona* and *Salix arctica* in Canadian High Arctic tundra [19]. Increased temperature (+1 °C, one growing season) has resulted in thinner leaves due to thinner epidermis, palisade and spongy layers in *Populus tremula* [20]. Warming has decreased the palisade-spongy ratio in *C. tetragona* (subarctic, +3~4 °C, 23 growing seasons) and *Empetrum nigrum* (Greenland tundra, +2.7 °C, two growing seasons) but has increased the thickness of the epidermis in *C. tetragona* and *Betula nana* (Greenland tundra, +2.7 °C, two growing seasons) [11,21]. 

Leaf anatomy showed acclimation to long-term warming. For example, Hartikainen et al. found that leaf anatomy can respond sensitively to short-term warming, but Schollert et al. showed no alterations of leaf anatomy to long-term warming [11,20]. The alterations in leaf anatomy can then affect photosynthetic gas exchange. For instance, increased leaf thickness and epidermis could be beneficial for water deficit and thus influence leaf physiology, and changes in palisade-spongy tissue ratio will change photosynthetic potential because palisade tissue has more chloroplasts [21].

To better understand and predict tundra species and ecosystem responses to future warming, we studied leaf photosynthesis, morphology and anatomy of three dominant tundra shrubs (*Dryas octopetala var. asiatica*, *Vaccinium uliginosum* and *Rhododendron confertissimum*) which have been artificially warmed by OTCs in situ on the Changbai Mountain tundra for 7 years. We hypothesized that a continuous 7 years of warming would stimulate photosynthesis at the individual scale, but with different magnitudes for different species. The changes in morphology and anatomy would benefit to improve photosynthesis. Therefore, we expected some species with higher responses in photosynthesis and/or morphology and/or anatomy to have competitive advantages over the other species.

## 2. Results

### 2.1. Environmental Measurements

The OTC treatment increased average air and soil temperatures, while the soil water content was decreased (Table 1). Compared to controls, OTCs increased the mean daily (24 h) air temperature during the growing season by 1.4 °C (2016) and 1.6 °C (2017). OTCs increased the daytime air temperature by 2.5 °C (2016) and 2.9 °C (2017), and the nighttime temperature by 0.9 °C (2016) and 0.3 °C (2017) (Table 1). Warming did not significantly affect daily soil temperature at 10 cm depth although there was an increased trend, 13.7 vs. 13.1 °C in 2016 and 13.9 vs. 12.9 °C in 2017. The daytime soil temperature was markedly increased by 2.9 °C in 2016 and 3.3 °C in 2017, but the nighttime soil temperature did not change as compared to the control (Table 1). The soil water content was decreased by the same scales during the day and the night.

### 2.2. The Light Response Curves 

**Figure 1:**
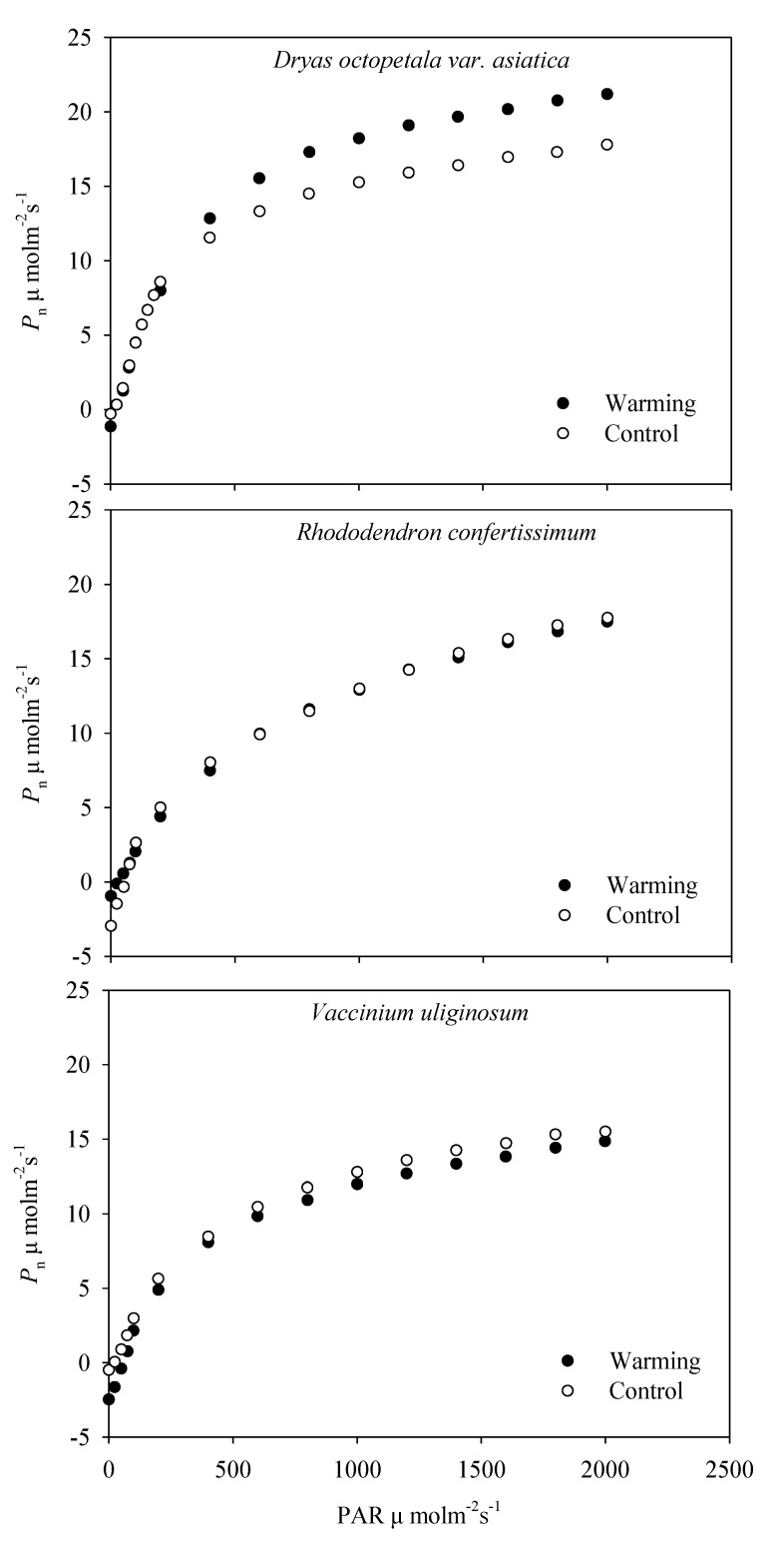
Light response curves of net photosynthetic rate (*P*_n_) in *Dryas octopetala* var. *asiatica*, *Rhododendron confertissimum* and *Vaccinium uliginosum* grown in the warming open-top chambers and control plots which were measured on 11 August 2017.

In all curves for *Dr. octopetala* var. *asiatica*, *V. uliginosum* and *Rh. confertissimum*, the measured and fitted photosynthetic rate showed linear responses with increasing PAR up to 200 μmol m^−2^ s^–1^, and the uptrend of the net photosynthetic rate slowed down after 1800 μmol m^–2^ s^–1^. As an example, Figure 1 showed the photosynthetic light-response curves measured on 11 August 2017. *Dr. octopetala* var. *asiatica* in the warming always showed a higher photosynthetic rate than that in the control. 

### 2.3. Photosynthetic Characteristics 

**Figure 2:**
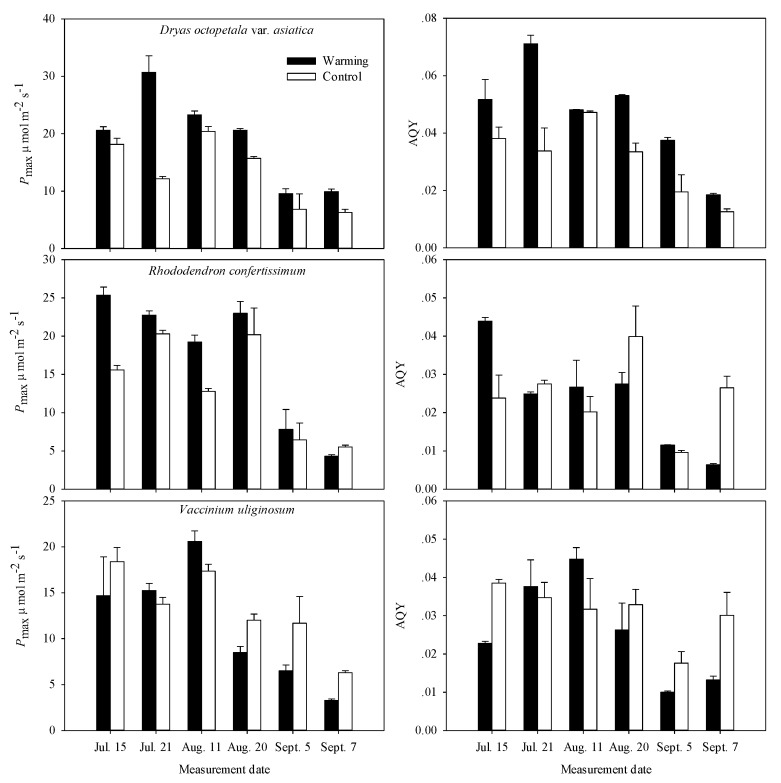
Light-saturated photosynthetic rates (*P*_max_) and apparent quantum yield (AQY) measured in *Dryas octopetala* var. *asiatica*, *Rhododendron confertissimum* and *Vaccinium uliginosum* grown in the warming open-top chambers and the control plots during the growing season (July to September) after 7 years’ warming treatment. In the figure, mean values are based on the individuals at least three chambers or control plots.

The *P*_max_ in both *Dr. octopetala* var. *asiatica* and *Rh. confertissimum* displayed significant differences between the warming and the control plots, and *P*_max_ in *Dr. octopetala* var. *asiatica* differed significantly between measurement dates. There were no significant interactions between treatment and measurement date on *P*_max_ for the three species (Table 2). Warming did not significantly affect the *P*_max_ in *V. uliginosum* (Figure 2, Table 2). On average, warming increased *P*_max_ by 43.6% for *Dr. octopetala* var. *asiatica* and by 26.7% for *Rh. confertissimum* across the whole growing season (Table 3). In addition, the higher *P*_max_ and marked increase in *P*_max_ mainly occurred in July and August (+21.0 to +68.4%). The photosynthetic rates declined rapidly at the end of growing season (September) for the three species (Figure 2). The *P*_max_ of *Dr. octopetala* var. *asiatica* was 1.5 times higher in OTCs (9.7 μmol m^−2^ s^−1^) than that in the controls (6.6 μmol m^−2^ s^−1^) in September. *Rh. confertissimum* grown in the warming and the control exhibited the same *P*_max_ of 6.0 μmol m^−2^ s^−1^ in September (Table 3). Smaller *P*_max_ of *V. uliginosum* in the warming OTCs compared to controls was observed at the end of the growing season (Figure 2). In addition, the *P*_max_ of *Dr. octopetala* var. *asiatica* and *Rh. confertissimum* was higher than that of *V. uliginosum* (Table 3).

The AQY of *Dr. octopetala* var. *asiatica* grown in the warming OTCs was 51.6% greater than that in the control plots across the whole growing season (Figure 2; Table 2 and Table 3). OTCs increased AQY in *Dr. octopetala* var. *asiatica* by 69.4% in July, 27.5% in August, and 7.5% in September compared to the control plots (Table 3). However, compared to the controls, *V. uliginosum* showed a marginally significant decrease in AQY (−16.1%; *p* = 0.044), while no changes in AQY were observed for *Rh. confertissimum* across the whole growing season (Table 2 and Table 3).

### 2.4. Leaf Anatomy

For *Dr. octopetala* var. *asiatica*, warming did not significantly change the leaf thickness (mean 136.3 μm for leaves both in the warming OTCs and the control plots) throughout the growing season (Table 4). Warming significantly decreased the leaf thickness by 26.6% for *Rh. confertissimum* in July (*p* = 0.022) and by 12.7% for *V. uliginosum* in September (*p* = 0.001). The three species showed relatively higher leaf thickness in July than in September for the warming and the control plots. The thickest leaves were found in *Rh. confertissimum* (mean 250.6 μm) and the thinnest in *Dr. octopetala* var. *asiatica* (mean 136.3 μm) (Table 4). As an example, Figure S1 showed the light microscopy images of leaf cross-sections for the three species measured on 11 August 2017.

There was no significant difference in palisade thickness (ranging from 63 μm to 70 μm) between the warming and the control plots for *Dr. octopetala* var. *asiatica*, *V. uliginosum* and *Rh. confertissimum* (all *p* > 0.05) (Table 4). Warming did not significantly affect the ratio of palisade to spongy parenchyma for the three species except for the measurements for *V. uliginosum* in September (Table 4). The ratios of palisade to leaf thickness and to spongy parenchyma in *Dr. octopetala* var. *asiatica* were the highest among the three species. 

Warming significantly decreased adaxial and abaxial epidermis cell length (−17.2~−29.8%) and cell thickness (−21.6~−39.6%) in *V. uliginosum*. However, warming significantly increased adaxial and abaxial epidermis cell length and thickness (+12.1~+17.9%) in *Rh. confertissimum* measured in July (Table 4). At the end of growing season (September), the cuticle thickness of *Rh. confertissimum* was 13.7% higher in the warming OTCs than that in the control plots (*p* < 0.05). The epidermis cell length and thickness in *Dr. octopetala* var. *asiatica* were not affected by warming (*p* > 0.05), except abaxial epidermis thickness measured in September. The cell length and thickness of *Dr. octopetala* var. *asiatica* were increased on average by 18.1 and 31.0% by warming during the growing season. The adaxial epidermis cells were bigger than abaxial epidermis for each species (Table 4).

### 2.5. Leaf Morphology

**Figure 3:**
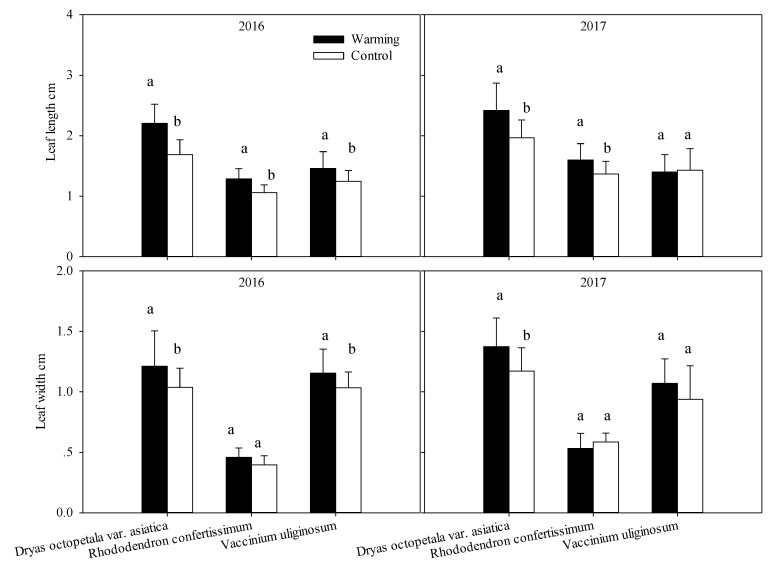
Leaf length and width in *Dryas octopetala* var. *asiatica*, *Rhododendron confertissimum* and *Vaccinium uliginosum* grown in the warming open-top chambers and the control plots measured during the growing seasons in 2016 and 2017. In the figure, mean values are those of 32 replicates and different lowercase letters indicate significant differences at the 0.05 level between the warming and the control for each species.

Across the two growing seasons, there were significant differences in leaf length and width between treatments for *Dr. octopetala* var. *asiatica* (Figure 3). Warming increased the leaf length by 29.4% in 2016 and 20.0% in 2017, and similarly increased leaf width by 20.0% in 2016 and 16.7 % in 2017 for *Dr. octopetala* var. *asiatica*. Warming increased the leaf length (+25.0%; *p* = 0.001) and width (+20.0%; *p* = 0.044) of *V. uliginosum* in 2016, but these increases disappeared in 2017. For *Rh. confertissimum*, warming affected the leaf length (+16%) only (Figure 3).

## 3. Discussions

Since plant growth in the tundra is mainly temperature limited, the marked increase in daytime temperature can promote photosynthesis in OTCs (Figure 2; Table 1 and Table 2). The relatively smaller increase in nighttime temperature is beneficial to the reduction of carbon consumption by respiration (Table 1). Thus, the environment of OTCs is expected to have positive effects on plants in the alpine tundra. However, the responses of leaf photosynthesis, morphology and anatomy to OTC warming for the three species in the present study are not always positive and show species-specific reactions. Previous studies also indicated that different species grown in the Antarctic were differently affected by warming [17,22]. For example, warming changed the photosynthesis and anatomical structure of *Colobanthus quitensis*, but it had no effects on those of *Deschampsia antarctica* [17,22]. These results suggest that measurements at the individual species level are still needed to better understand and predict the responses at the community and ecosystem level of tundra vegetation under future climate warming.

The photosynthesis of high alpine plants have been found to increase [9,23], decrease [23] or not change [24] in response to climate warming. In the present study, the *P*_max_ of *Dr. octopetala* var. *asiatica* was significantly increased by warming. *Rh. confertissimum* also showed increases in *P*_max_, but the magnitude of stimulation was smaller than in *Dr. octopetala* var. *asiatica*. By contrast, warming tended to reduce *P*_max_ of *V. uliginosum.* We also found increased leaf size in 2016 had not been maintained in 2017 for *V. uliginosum*. Thus, *V. uliginosum* probably acclimated to long-term warming treatment. Similarly, Carroll et al. also found different responses of photosynthesis and leaf size to warming in three dominant tree species (*Pinus contorta* var. *latifolia*, *Pinus ponderosa* and *Populus tremuloides*) [25]. Long-term responses of photosynthesis to changes in temperature can cause a shift in optimum temperature of leaf photosynthesis, which stimulates photosynthesis at the new growth temperature, or changes the shape of the photosynthesis-temperature curve without shifting optimum temperature [26,27]. Plants growing in the cool region can also increase the activity of photosynthesis-related enzymes to acclimate to warming, thereby enhancing photosynthesis [27]. 

Photosynthesis and morphology exhibit comparatively strong plasticity to warming. Leaf anatomical features are relatively less susceptible to environmental change, and therefore the variations in anatomy might reflect long-term adaption to warming. We asked whether the differential responses of photosynthesis to long-term warming related to specific morphology and anatomical adjustments. No observed anatomical determinants of *Dr. octopetala* var. *asiatica* were affected by warming compared to the controls. However, the leaf size of *Dr. octopetala* var. *asiatica* was significantly stimulated, which means the total photosynthetic area and photosynthate increased. By contrast, the photosynthetic response of *Colobanthus quitensis* in the Antarctic to warming relied on specific adjustments in the anatomical determinants, which enhanced photosynthetic assimilation, thereby promoting plant growth [17]. 

Among the leaf anatomical traits, palisade tissue might be the most important to photosynthesis because palisade mesophyll cells are the structural powerhouse of photosynthesis. The thickness of palisade mesophyll has been found to be positively correlated with net photosynthetic rate [21]. However, both the *P*_max_ and AQY of *Dr. octopetala* var. *asiatica* were significantly increased, which is inconsistent with the anatomical traits. This indicates that photosynthesis, at least for *Dr. octopetala* var. *asiatica,* does not integrate closely to anatomical characteristics, which does not support our hypothesis. Photosynthesis is relatively sensitive to environmental factors, like temperature, light and water status. The activity of photosynthesis-related enzyme increases with temperature, which will stimulate photosynthesis but not necessarily cause changes in anatomical structure. 

We found increases in leaf size of *Dr. octopetala* var. *asiatica* and *Rh. Confertissimum* in response to long-term warming. The leaf area and growth are determined by the fraction of photosynthate required for plant respiration. In the present study, increased leaf size, photosynthesis and number of individuals (32% in the warming OTCs vs. 25% in the control plots) of *Dr. octopetala* var. *asiatica* in OTCs imply significant advantages of this species in a warming world. Larger leaves in shrubs of *Cassiope tetragona*, *Salix arctica* and *Dr. integrifolia* were also observed in the Canadian High Arctic after 16 years of warming treatment (+1~2 °C) [19]. Similar palisade thickness but relatively thinner leaf thickness in *Dr. octopetala* var. *asiatica* than in *Rh. confertissimum* and *V. uliginosum* (Table 4) suggests that *Dr. octopetala* var. *asiatica* will grow better than the other two species if the Changbai Mountain tundra continues warming. Taken together, our results suggest that global warming will benefit *Dr. octopetala* var. *asiatica* in the tundra on Changbai Mountain. In the long term, warming favors greater dominance by *Dr. octopetala* var. *asiatica*. Based on differential responses of photosynthesis and leaf size to warming among the three dominant tree species (*Pinus contorta* var. *latifolia*, *Pinus ponderosa* and *Populus tremuloides*) in a community, Carroll et al. concluded that forest composition will be altered in a future warming world [25]. Thus, we may also predict that future warming might promote expansion and enhance the cover of *Dr. octopetala* var. *asiatica*, whereas *V. uliginosum* may be at risk for gradual occupation by other species, which could lead to changes in the tundra ecosystem function on Changbai Mountain.

Thinning of the leaf and thickening of epidermis cells in *Rh. confertissimum* by warming probably imply less diffusion space of CO_2_ in spongy parenchyma. Similar results were observed by Schollert et al. that the epidermis of *Betula nana* in Greenland was also thickened by OTC warming [21]. The cuticle thickness of *Rh. confertissimum* in the warming was higher than the control in September (Table 4). This situation in *Rh. confertissimum* is not beneficial for photosynthetic carbon uptake but probably good for water conservation in the long term if the climate continues to warm. Bacelar et al. reported that a thicker epidermis (including cuticle) was an anatomical adaptation to improve water conservation [28]. A thicker epidermis in response to warming may be a structural adaptation for prevention of water loss. However, a significant decrease in adaxial and abaxial epidermis thickness with decreased leaf thickness in *V. uliginosum* under warming was also observed in the present study. Similar results of decreased epidermis thickness to warming have been found in some boreal forest species such as *Pinus sylvestris* and *Populus tremula* [20,29]. For *Dr. octopetala* var. *asiatica*, warming had no effects on the epidermis thickness and other leaf anatomical traits, which is in accordance with results in *Empetrum hermaphroditum* [11]. 

No significant changes in palisade-spongy parenchyma ratio have been observed for the three species, but with an increased trend in *Dr. octopetala* var. *asiatica* and *Rh. confertissimum*, and decreased trend in *V. uliginosum*. Coincidentally, warming increased the *P*_max_ of *Dr. octopetala* var. *asiatica* and *Rh. confertissimum* and caused a decline in *P*_max_ of *V. uliginosum*. A decreased palisade-spongy parenchyma ratio has also been observed for *Betula nana* and *Cassiope tetragona* under warming [11]. The higher palisade-spongy parenchyma ratio suggests a compact arrangement of cells and high mesophyll surface area which could facilitate CO_2_ uptake and thus maintain higher photosynthesis [30].

## 4. Materials and Methods

### 4.1. Study Site and Experimental Design

The study was conducted in the tundra ecosystem at 2028 m a.s.l. on the north-facing slope of Changbai Mountain (41°58′–42°42′ N; 127°67′–128°27′ E), northeastern China. The experimental site has a mean annual temperature of −7.3 °C and mean annual precipitation of 1373 mm [31,32]. A snow-free season lasts from June to August (growing season). The average growing season (June to September) temperature is 5.87 °C and average precipitation ranges from 700 to 1400 mm [33]. The vegetation in the experimental plots is dominated by *Dr. octopetala* var. *asiatica*, *V. uliginosum* and *Rh. confertissimum.* After 7 years of OTC warming in 2017, the average coverage was 32% (OTCs) and 21% (controls) for *Dr. octopetala* var. *asiatica*, 32% (OTCs) and 25% (controls) for *V. uliginosum*, and 6% (OTCs) and 9% (controls) for *Rh. confertissimum*. 

Eight hexagonal clear-sided OTCs, according to the criteria of the International Tundra Experiment [3], were installed in the tundra in 2010. The OTCs were 45 cm high, had inclined sides (60 °C), enclosed a surface of 1.0 m^2^, and were left in the experimental plots year round. The OTCs were installed on relatively flat ground with similar species composition and vegetation coverage. Equal areas of control plots with similar characteristics were adjacently established to each OTC. The spatial arrangements of OTCs and control plots were randomly based on the similarity of the vegetation. 

The Em 50 Data Collection System (Decagon Devices Inc., Pullman, WA, USA) was respectively placed in each OTC and the corresponding control plot. The air temperature at the height of 15 cm above the ground surface, soil temperatures at 10 cm soil depth, air and soil relative humidity, and photosynthetically active radiation (PAR) were monitored and recorded every half an hour. All sensors were put in the research field only during growing seasons (June to September). We separated daytime from 0600 h to 1800 h and nighttime from 1800 h to next 0600 h to distinguish different warming effects between daytime and nighttime since daytime warming is more effective for photosynthesis. We showed only the data recorded during the growing seasons in 2016 and 2017. 

### 4.2. Photosynthetic Light Response Curves

Photosynthesis was measured under ambient CO_2_ concentration (approx. 350 μmolmol^−1^) using LI-6400 portable photosynthesis systems with a red/blue LED light source (Li-Cor Inc., Lincoln, Dearborn, MI, USA) between 0900 h and 1130 h on sunny days. All measurements were conducted at ambient temperature to evaluate seasonal differences. At least three replicate measurements were made for each species in the warming OTCs and control plots on 21 and 28 July, 11 and 30 August, 5 and 7 September 2017. Leaf chamber temperature was maintained at ambient temperature without temperature control, representing the natural temperature experienced by leaves during the measurement time. Relative humidity inside the leaf chamber ranged from 30% to 50%. Net photosynthetic rate was determined at a series of light levels of photosynthetically active radiation (PAR) of 0, 25, 50, 75, 100, 125, 150, 175, 200, 400, 600, 800, 1000, 1200, 1400, 1600, 1800, and 2000 μmol m^−2^ s^−1^. After leaf dark acclimation, PAR was increased gradually from 0 to each light level needed, and the net photosynthetic rates (*P_n_*) were manually recorded when stabilized. The slope of the linear part of the light-response curve when PAR was between 0 and 200 μmol m^−2^ s^−1^ was defined as apparent quantum yield (AQY). To estimate theoretically the maximal light-saturated photosynthetic rate (*P*_max_), light response curves were modeled by fitting non-rectangular hyperbola described by Prioul and Chartier [34].

$$P_{N}=\frac{\alpha PAR+P_{\max}-\sqrt{{\left(\alpha PAR+\;P_{\max}\right)}^{2}-4\theta \alpha PARP_{\max}}}{2\theta }-R_{d}$$

where *P*_max_ is the maximum light-saturated net photosynthetic rate (μmol CO_2_ m^−2^ s^−1^), PAR is (μmol m^−2^ s^−1^) the photosynthetically active radiation, α is the initial slope or AQY, and *θ* is the convexity or curvature factor (between 0 and 1). *R_d_* is the rate of respiration in the light. *P*_max_ was estimated by the above formula in the present study. The fitted α based on the above formula was far higher than the initial slope of the measured curve, so we used the initial slope during PAR < 200 μmol m^−2^ s^−1^ as AQY.

### 4.3. Leaf Morphology Measurement

Thirty-two plants per species (*Dr. octopetala* var. *asiatica*, *Rh. confertissimum* and *V. uliginosum*) were selected to measure leaf length and width by digital caliper (four individuals per OTC and control plot) during the growing seasons in 2016 and 2017. Leaf width was measured at the widest part of a leaf. 

### 4.4. Leaf Anatomy Measurement

Three leaves from three randomly selected plants per species from each OTC and control plot were collected on 13 July and 18 September 2017. All leaves were immediately fixed in FAA (5 mL 38% formaldehyde, 90 mL 70% ethanol, and 5 mL acetic acid in a proportion of 1:18:1 *v*/*v*) after cutting down. Eight leaves from the warming OTCs and eight leaves from the control were selected at random to make a paraffin section for one sampling date. Each leaf had two pieces avoiding midribs and margins, and each transversal section was observed by three views. The leaf anatomy characteristics were observed with a light microscope and photographed (Motic BA 300) with a 100 magnification. Leaf thickness was measured with complete structure and was estimated as an average of at least three points. Thicknesses of palisade tissues were measured at the same point as leaf thickness for both warming and the control plots [11]. 

### 4.5. Statistical Analysis

The normality of the distribution and homogeneity of the data were checked (Kolmogorov–Smirnov test) before any statistical analyses. One-way ANOVA was used to test the differences in environmental factors. After significant regression relationships between net photosynthetic rate and PAR less than 200 μmol m^−2^ s^−1^, the slope of the linear part represented AQY. Repeated measures ANOVA was used to assess the effects of warming and measurement date on *P*_max_, AQY and anatomic parameters. Leaf length and width were evaluated by one-way ANOVA with treatment as the main factor. All statistical analyses were conducted with SPSS 16.0 system (SPSS Inc., Chicago, IL, USA) and Excel (2013). All tests of statistical significance were conducted at a level of 0.05.

## 5. Conclusions

Our results indicate that in response to warming, the magnitude and direction of leaf photosynthesis, morphology and anatomy differ with species. *Dr. octopetala* var. *asiatica* showed an obvious advantage in *P*_max_, leaf size and relatively higher palisade proportion than *Rh. confertissimum* and *V. uliginosum*; therefore, we may expect that *Dr. octopetala* var. *asiatica* population could hold greater dominance on the Changbai Mountain tundra with the future continuous warming, and *Rh. confertissimum* and *V. uliginosum* might eventually decrease. Species-specific responses of leaf physiological traits to warming suggest that the measurements at the individual species level are still needed to better understand and predict the community- and/or ecosystem-level responses to future climate change and their responses in high latitude and high altitude ecosystems.

## Figures and Tables

**Table 1:** Air temperature, air relative humidity, soil temperature at 10 cm depth, and soil water at the scale of daily, daytime and nighttime during the growing seasons in 2016 and 2017.

Parameter		2016		Significance		2017		Significance
	OTCs	Controls	Difference		OTCs	Controls	Difference	
Daily air temperature (°C)	15.3	13.7	+1.6	**	13.4	12.0	+1.4	**
Daily relative humidity (%)	85	84	+1	ns	79	77	+2	ns
Daily soil temperature at 10 cm depth (°C)	13.7	13.1	+0.6	ns	13.9	12.9	+1.0	ns
Daily soil water content (m^3^ m^−3^)	0.314	0.358	−0.044	***	0.257	0.309	−0.052	***
Daytime air temperature (°C)	18.7	16.2	+2.5	***	16.0	13.1	+ 2.9	***
Daytime relative humidity (%)	78	81	3	ns	72	73	1	ns
Daytime soil temperature at 10 cm depth (°C)	17.5	14.6	+2.9	***	17.8	14.5	−3.3	***
Daytime soil water content (m^3^ m^−3^)	0.313	0.358	−0.045	***	0.257	0.309	−0.052	***
Nighttime air temperature (°C)	12.1	11.2	+0.9	*	10.9	10.6	+0.3	*
Nighttime relative humidity (%)	91	86	+5	ns	86	80	+6	ns
Nighttime soil temperature at 10 cm depth (°C)	11.3	11.3	0	ns	11.0	11.2	−0.2	ns
Nighttime soil water content (m^3^ m^−3^)	0.315	0.358	−0.043	***	0.257	0.309	−0.052	***
Daytime PAR (μmol m^−2^ s^−1^)	655	676	21	ns	722	768	−46	ns

Notes: We artificially separated daytime from 0600 h to 1800 h and nighttime from 1800 h to next 0600 h. *** *p* < 0.001; ** *p* < 0.01; * *p* < 0.05; ns *p* > 0.05.

**Table 2:** Statistical results of the effects of warming treatment and measurement date on photosynthetic parameters of *Dryas octopetala* var. *asiatica*, *Rhododendron confertissimum* and *Vaccinium uliginosum*. *P*_max_, light-saturated photosynthetic rate. AQY, apparent quantum yield.

	*Dr. octopetala* var. *asiatica*		*Rh. confertissimum*		*V. uliginosum*	
	*P*_max_	AQY	*P*_max_	AQY	*P*_max_	AQY
Treatment	**	*	***	ns	ns	*
Measurement date	*	*	ns	ns	ns	ns
Interaction	ns	ns	ns	ns	ns	ns

Notes: Significance * *p* < 0.05, ** *p* < 0.01, *** *p* < 0.001, ns *p* > 0.05.

**Table 3:** The maximal light-saturated photosynthetic rate (*P*_max_) and apparent quantum yield (AQY) derived from light-response curves for *Dryas octopetala* var. *asiatica*, *Rhododendron confertissimum* and *Vaccinium uliginosum* grown in the warming open-top chambers and the control plots during the whole growing season. The values in the table are the monthly means based on the data in Figure 2.

	July		August		September		Whole Growing Season	
	Warming	Control	Warming	Control	Warming	Control	Warming	Control
*Dryas octopetala* var. *asiatica*								
*P*_max_ (μmol m^−2^ s^−1^)	25.6	15.2	21.9	18.1	9.7	6.6	19.1	13.3
AQY	0.061	0.036	0.051	0.040	0.028	0.016	0.047	0.031
*Rhododendron confertissimum*								
*P*_max_ (μmol m^−2^ s^−1^)	24.0	18.0	21.1	16.5	6.1	6.0	17.1	13.5
AQY	0.034	0.026	0.027	0.030	0.009	0.018	0.023	0.025
*Vaccinium uliginosum*								
*P*_max_ (μmol m^−2^ s^−1^)	15.0	16.1	14.5	14.7	4.9	9.0	11.5	13.2
AQY	0.030	0.037	0.036	0.032	0.012	0.024	0.026	0.031

Notes: Limited sample size of two in each month prohibited error to be calculated for *P*_max_ and AQY.

**Table 4:** Leaf anatomy variables for *Dryas octopetala* var. *asiatica*, *Rhododendron confertissimum* and *Vaccinium uliginosum* collected on 13 July and 18 September 2017 in the open-top chambers and control plots. The different lowercase letters in the same row indicate significant differences at the level of 0.05. (Means ± SE, *n* = 24).

	Warming		Control	
	July	September	July	September
*Dryas octopetala* var. *asiatica*				
Leaf thickness (μm)	147.2 ± 3.033 a	125.4 ± 2.560 b	150.0 ± 3.131 a	122.5 ± 3.401 b
Palisade thickness (μm)	67.7 ± 2.413 a	65.7 ± 2.528 a	69.2 ± 1.475 a	62.6 ± 1.341 a
Palisade: leaf thickness	0.459 ± 0.012 b	0.525 ± 0.019 a	0.463 ± 0.007 b	0.513 ± 0.011 a
Palisade: spongy parenchyma	1.343 ± 0.114 b	1.790 ± 0.195 a	1.241 ± 0.044 b	1.532 ± 0.101 ab
Adaxial epidermis length (μm)	21.0 ± 0.712 a	17.3 ± 0.609 b	21.3 ± 0.638 a	16.9 ± 1.009 b
Adaxial epidermis thickness (μm)	13.8 ± 0.520 a	11.2 ± 0.647 b	12.9 ± 0.473 a	9.7 ± 0.537 b
Abaxial epidermis length (μm)	13.5 ± 0.461 a	12.3 ± 0.856 ab	13.0 ± 0.387 ab	11.3 ± 0.931 b
Abaxial epidermis thickness (μm)	10.2 ± 0.315 a	8.2 ± 0.508 b	9.9 ± 0.326 a	6.9 ± 0.222 c
*Rhododendron confertissimum*				
Leaf thickness (μm)	254.3 ± 10.335 b	222.8 ± 11.513 c	280.9 ± 6.895 a	244.5 ± 7.164 bc
Palisade thickness (μm)	63.5 ± 2.933 a	65.9 ± 2.796 a	69.9 ± 2.890 a	70.8 ± 2.164 a
Palisade: leaf thickness	0.252 ± 0.007 b	0.301 ± 0.012 a	0.247 ± 0.006 b	0.293 ± 0.008 a
Palisade: spongy parenchyma	0.404 ± 0.020 b	0.482 ± 0.031 a	0.372 ± 0.012 b	0.478 ± 0.022 a
Adaxial epidermis length (μm)	20.9 ± 0.583 a	17.0 ± 0.545 b	18.1 ± 0.494 b	17.8 ± 0.680 b
Adaxial epidermis thickness (μm)	14.2 ± 0.465 a	9.3 ± 0.429 d	12.7 ± 0.309 b	10.7 ± 0.397 c
Abaxial epidermis length (μm)	16.0 ± 0.557 a	12.6 ± 0.863 b	13.6 ± 0.389 b	12.4 ± 0.431 b
Abaxial epidermis thickness (μm)	12.2 ± 0.626 a	8.6 ± 0.368 c	10.5 ± 0.301 b	8.9 ± 0.352 c
Cuticula thickness (μm)	7.1 ± 0.269 b	8.5 ± 0.542 a	6.8 ± 0.225 b	7.5 ± 0.250 b
*Vaccinium uliginosum*				
Leaf thickness (μm)	174.8 ± 2.766 a	149.5 ± 4.412 b	180.8 ± 4.218 a	171.2 ± 4.445 a
Palisade thickness (μm)	66.3 ± 1.451 ab	67.0 ± 3.013 ab	70.5 ± 2.153 a	64.0 ± 2.233 b
Palisade: leaf thickness	0.381 ± 0.007 b	0.448 ± 0.014 a	0.391 ± 0.009 b	0.375 ± 0.011 b
Palisade: spongy parenchyma	0.793 ± 0.029 b	1.008 ± 0.095 a	0.886 ± 0.036 ab	0.773 ± 0.059 b
Adaxial epidermis length (μm)	21.8 ± 0.710 b	24.0 ± 1.104 b	29.2 ± 0.980 a	29.0 ± 1.113 a
Adaxial epidermis thickness (μm)	12.3 ± 0.475 b	8.1 ± 0.646 c	16.0 ± 0.576 a	12.3 ± 0.594 b
Abaxial epidermis length (μm)	17.8 ± 0.640 b	16.3 ± 1.087 b	22.0 ± 0.738 a	23.2 ± 0.703 a
Abaxial epidermis thickness (μm)	9.8 ± 0.280 b	6.4 ± 0.618 c	12.5 ± 0.515 a	10.6 ± 0.611 b

## Supplementary Materials

The following are available online at https://www.mdpi.com/2223-7747/8/8/271/s1, Figure S1: Light microscopy images of leaf cross-sections for *Dryas octopetala* var. *asiatica* in the warming OTCs (a) and in the ambient control (b), *Vaccinium uliginosum* in the warming OTCs (c) and in the control (d), *Rhododendron confertissimum* in the warming OTCs (e) and in the control (f), sampled in July 2017 (measured data in detail in Table 4).
