# Supplementary material for: Leaf Anatomy, Morphology and Photosynthesis of Three Tundra Shrubs after 7-Year Experimental Warming on Changbai Mountain
Source: Plants (Basel). 2019 Aug 7;8(8):271. doi: 10.3390/plants8080271 (PMC6724111; doi:10.3390/plants8080271)
Supplement: Supplementary file 1 [file plants-08-00271-s001.pdf]

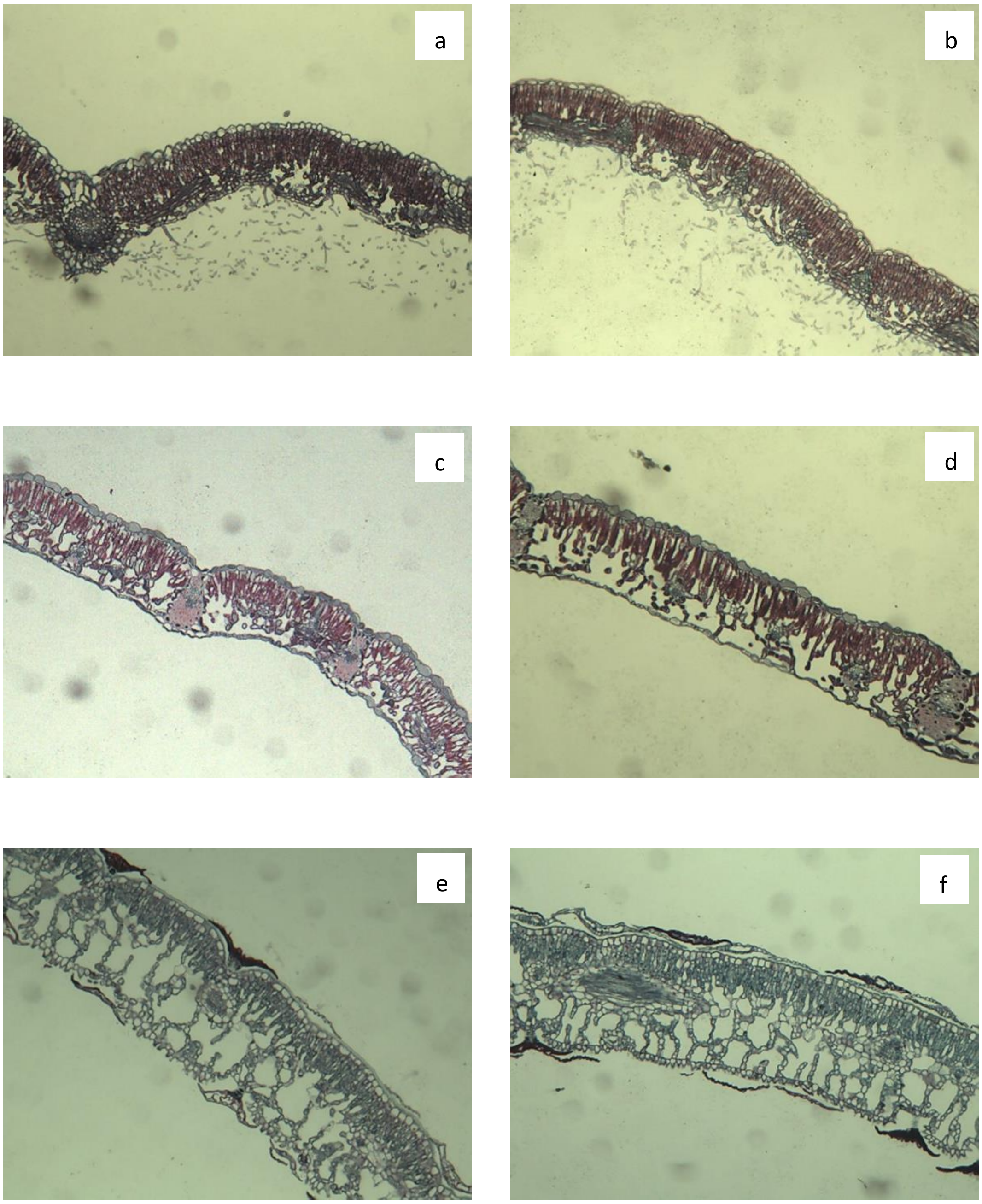

Figure S. Light microscopy images of leaf cross-sections for *Dryas octopetala* var. *asiatica* in the warming OTCs (a) and in the ambient control (b), *Vaccinium uliginosum* in the warming OTCs (c) and in the control (d), *Rhododendron confertissimum* in the warming OTCs (e) and in the control (f), sampled in July 2017 (measured data in detail in Table 4).
